# Effect of Olive Pit Reinforcement in Polylactic Acid Biocomposites on Environmental Degradation

**DOI:** 10.3390/ma16175816

**Published:** 2023-08-24

**Authors:** Sofía Jurado-Contreras, Francisco J. Navas-Martos, José A. Rodríguez-Liébana, M. Dolores La Rubia

**Affiliations:** 1 Andaltec Technological Centre, Ampliación Polígono Industrial Cañada de la Fuente, C/Vilches 34, 23600 Martos, Spain; sofia.jurado@andaltec.org (S.J.-C.); francisco-javier.navas@andaltec.org (F.J.N.-M.); jose-antonio.rodriguez@andaltec.org (J.A.R.-L.); 2 Department of Chemical, Environmental and Materials Engineering, Campus Las Lagunillas, University of Jaén, 23071 Jaén, Spain; 3 University Institute of Research on Olive and Olive Oils (INUO), Campus Las Lagunillas, University of Jaén, 23071 Jaén, Spain

**Keywords:** olive pit, polylactic acid, biocomposites, degradation

## Abstract

Polylactic acid (PLA) is a biomaterial widely used as an alternative to petroleum-based polymeric matrices in plastic components. PLA-based biocomposites reinforced with lignocellulosic waste are currently receiving special attention owing to their mechanical properties, low toxicity, recyclability, and biodegradability. The influence of the percentage of waste on their properties and resistance to degradation are some of the points of great relevance. Therefore, a series of PLA-based biocomposites containing different percentages of olive pits (5, 15, 25 and 40% wt.) were manufactured and characterized both (a) immediately after manufacture and (b) after one year of storage under environmental conditions. The results obtained were analyzed to evaluate the influence of the incorporation of olive pits on the resistance to degradation (measured through Carbonyl Indices, CI), mechanical properties (tensile, flexural and impact strength), structure (Fourier Transform Infrared Spectroscopy, FT-IR; and, X-ray Diffraction, XRD), morphology (Scanning Electron Microscopy, SEM) and water absorption capacity of the manufactured materials. PLA degradation, corroborated by Differential Scanning Calorimetry (DSC), FT-IR, and XRD, resulted in a decrease in tensile and flexural strengths and an increase in the tensile and flexural moduli. This trend was maintained for the biocomposites, confirming that reinforcement promoted the PLA degradation.

## 1. Introduction

Globally, 140 million tons of fossil-origin polymers are generated annually. The production of this type of plastic results in a large amount of wastage. In 2050, the annual production of 850 million tons of this waste is estimated [1]. Green biocomposites play a key role in reducing environmental problems related to plastic waste [2]. Polylactic acid (PLA) is one of the most widely produced bioplastics worldwide. Although this polymer is used in high-end applications, it is expensive to produce. Advancements in the production of PLA from corn starch and sugar cane have led to a reduction in its cost and increased its possible applications, such as in automotive components, food packaging, and the textile industry, among others. PLA has interesting properties such as biocompatibility, elasticity, thermoplastic behavior, good moldability, and rigidity [1,3]. However, it also has some limitations such as high brittleness, sensitivity to enzymatic and environmental degradation, and low resistance to impact [4].

The introduction of reinforcements of lignocellulosic origin is a practice for improving the mechanical, physical, and thermal properties of PLA without compromising its design and manufacturing capacity [4]. In addition, natural fibers, such as those derived from cotton, linen, jute, hemp and oil palm empty fruit brunches, are becoming a trend in the replacement of synthetic fibers, thus producing fully biodegradable manufactured polymer composite products and reducing manufacturing costs [3,5].

Olive pit (OP) is the most abundant residue in olive oil production [6]. Typically, olive pits are used to obtain energy via combustion [7]. Studies on the benefits of using OP as reinforcement for thermoplastic matrices such as polystyrene [8], polyethylene [9], polypropylene [10,11] and poly(ε-caprolactone) [12] have been previously reported. Regarding PLA/OP biocomposites, Koutsomitopoulou et al. (2014) studied the effect of incorporating OP in a range from 0 to 20% wt. in a PLA matrix with different particle sizes [7]. Perinovic et al. (2010) studied the influence of the incorporation of OP on the thermal properties of poly(L-lactide) matrices, and observed a decrease in thermal stability after the incorporation of OP [13]. These studies showed the same trend as a function of the increase in the OP content in the biocomposite: an increase in the elastic modulus with a consequent decrease in the tensile strength and elongation at break, which is closely related to an increase in the cavities formed in the composite and poor interfacial adhesion [6,7].

Numerous elements can participate in the decomposition of PLA, including enzymes, water, ultraviolet radiation and microbes [1,14]. Under environmental conditions, two mechanisms mainly govern PLA degradation: hydrolysis and photolysis. When exposed to moisture, soluble monomers are released after cleavage of the ester groups from their main polymer chain, leading to a reduction in the molecular weight [1,15]. The breaking of the ester bonds begins when the water molecules enter the amorphous zones of the polymer and continue to advance to the outer layer of the crystalline zones. This leads to changes in the mechanical and thermal properties, morphology, and molecular weight of PLA [15], hence leading to a decrease in tensile strength, glass transition temperature, and elongation [1,16]. In photooxidative degradation, the carbonyl group of PLA absorbs UV radiation below 280 nm. During this degradation process, dissociation of the C-O bond and subsequent formation of carboxylic acids and anhydrides occurs [17].

Materials derived from nature, such as PLA and lignocellulosic reinforcements, are more sensitive to degradation processes than conventional plastics [4]. Some lignocellulosic fillers show peculiar characteristics that could be crucial for improving the biodegradability of such plastics, increasing the hydrophilicity of the composite due to the presence of channels and, therefore, promoting hydrolytic degradation [2]. However, the presence of natural fibers modifies the properties of the material when faced with degradation, as it increases the roughness of the surface and facilitates degradation. The degradation rate of polymer-based materials depends mainly on the surface state. A polymer with a rough surface degrades more easily than a polymer with a lower roughness. Szaraniec and Goryczka (2010) studied the deterioration in the mechanical properties of PLA/hemp and PLA/jute composites after soaking in water, and concluded that the materials were more sensitive to hydrolytic degradation than biodegradation [18]. Similarly, Chaturvedi et al. (2022) studied the influence of the type of reinforcement (walnut shell or pine needle ash) on the hydrolytic degradation process of PLA, thus reporting a decrease in the mechanical properties (tensile strength and elongation at break) [3].

The removal of polymer-based materials after their useful life is a key factor in reducing the environmental impact of bioplastics, such as PLA [1]. Therefore, it is vital to study the degradation mechanisms of composites based on this polymer matrix [3,4]. This study aimed to evaluate the behavior of PLA reinforced with OP and its influence on the degradation process when exposed to environmental temperature and humidity for one year.

The main novelties of this research work are the following: (a) the study focused on the analysis of the influence of the percentage by weight of OP on the degradation process of the materials as a consequence of their storage for one year in controlled environmental conditions of temperature and humidity; (b) a single range of sizes of the OP particle were considered, specifically that resulting from the milling process, leaving out the size classification process and simplifying the number of steps to obtain the composites; (c) additional additives were used to improve processability and phase compatibility, with the aim of making the manufacturing process and biocomposites more efficient; (d) the range of percentages by weight of OP of the manufactured biocomposites was twice as wide as that reported by other researchers; (e) in addition to the mechanical and morphological properties of the biocomposites, their thermal, structural and water absorption properties were analysed.

## 2. Materials and Methods

The OP was supplied by local suppliers located in Jaén Province (Spain). It was received once the process of obtaining olive oil was carried out, containing remains from the end of this process, such as rests of pulp and olive skin.

Ingeo™ Biopolymer 3251D PLA was supplied in pellet form by NatureWorks (Minnetonka, MN, USA). As indicated by the manufacturer, the material has a density of 1.24 g/cm^3^, a melt flow index of 35 g/10 min at 190 °C and 2.16 kg load, and a glass transition temperature (*T_g_*) of 55–60 °C. Two additives were used: (a) BYK P-MAX, a process additive based on a fatty acid ester (BYK-Chemie GmbH, Offenbach am Main, Germany) as a powder, and (b) Excellor PO1020, an additive with a high level of maleic anhydride grafting (MAPP) in the form of pellets (Exxon, Irving, TX, USA).

### 2.1. Conditioning and Characterization of OP

The OP received was subjected to successive washing cycles to eliminate non-valuable components (pomace and olive skin remains) and was dried in an oven at 60 °C. After drying, the samples were shredded using an ultra-centrifugal mill ZM 200 (RETSCH, Haan, Germany) to obtain the final OP with a suitable particle size range. This equipment achieved an adequate reduction in the size of the OP particles in a single pass by means of a rotor-ring sieving system. Specifically, a stainless steel ring sieve with a pore size of 0.5 mm was used. A rotation speed of 18,000 rpm was used to obtain a 0.5 L sample after 5 min of grinding.

The particle size of OP was determined by using a Mastersizer 2000 particle analyzer (Malvern Panalytical, Malvern, UK). To study morphology, Scanning Electron Microscopy (SEM) was performed using JSM 840 equipment (JEOL, Tokyo, Japan) that operated at a voltage of 15 kV. To identify the main chemical functional groups, attenuated total reflection (ATR) analysis was performed in conjunction with Fourier Transform Infrared Spectroscopy (FT-IR) using Vertex 70 equipment (Bruker, MA, USA). FT-IR spectra were recorded in the wavenumber range of 4000–400 cm^−1^. The chemical composition of the OP, in terms of cellulose, hemicellulose, and lignin, was determined by following the methodology proposed by Browning [19]. After hydrolysis of OP with concentrated sulfuric acid, the sugar content (D-glucose, D-xylose, and L-arabinose) was analyzed using a high-performance liquid chromatography (HPLC) system (TSQ Quantum Access Max, Thermo Fisher Scientific, Waltham, MA, USA). X-ray Diffraction (XRD) was also used to evaluate the structure and the crystalline index of OP. The measurements were performed in an Empyrean equipment with a PIXcel-3D detector (Malvern Panalytical, UK) in the 2θ interval from 10° to 60° with a step size of 0.02. Finally, the thermal stability of the OP was studied by using a thermogravimetric analyzer TGA Q500 (TA Instruments, New Castle, DE, USA). The OP was heated from 25 to 700 °C at a heating rate of 10 °C/min under N_2_ flow to obtain the weight loss curve (TGA, conventional thermogram) and its derivative (DTG, differential thermogram).

### 2.2. Manufacture of Biocomposites

To determine the influence of the OP on the properties of the PLA matrix, a series of biocomposites was manufactured with different percentages of reinforcement (0, 5, 15, 25, and 40% by weight). All biocomposites were manufactured with 1.5% wt. of BYK and 4% wt. of PO1020. The influence of these additives on a thermoplastic matrix has been previously studied [20]. Table 1 shows the compositions of the five different manufactured PLA-based biocomposite materials.

To identify the effect of the OP on the degradation of the PLA and its properties, all the manufactured biocomposites were subjected to a degradation process for one year. The denomination of the biocomposites after the degradation process (PLAD-based biocomposites) is presented in Table 2.

Prior to manufacturing the biocomposites, the conditioned OP was dried in an oven at 65 °C for 24 h to remove moisture. PLA pellets were dried at 60 °C for 10 h using a KKT 75 dryer (Werner Koch Maschinentechnik GmbH, Isprung, Germany). Finally, the compounding process was carried out on a Mini E-Lab 22 extrusion-pelletizing production line (Eurexma, Tradate, Italy) to obtain biocomposites in the form of pellets. The parameters of the compounding process are listed in Table 3.

Inside the extruder, the melting processes of the polymeric matrix and the additives and their intimate mixing with OP particles take place in zones I–VI. This is achieved by means of an adequate selection of the temperatures of each one of the zones, speed of rotation of the screws and suitable feeding of the components. The polymer-based materials are fed and advanced through the extruder chamber as a result of the rotation of the screws at the same time that they begin to melt. Once melted, the configuration of the screws allows an intimate mixture of the fluid phases between them and with the solid particles of OP, so that at the end of the residence time of the components inside the extruder chamber a proper mixing of the components makes it possible to obtain a biocomposite thread ready to be granulated and transformed into pellets at the output of the equipment.

### 2.3. Characterization of the Resulting Biocomposites

#### 2.3.1. Manufacture of Specimens

The required test specimens were obtained using injection molding technology in a Victory 28 injection machine (ENGEL, Schwertberg, Austria), removing moisture from the pellets in a KKT 75 dryer (Werner Koch Maschinentechnik GmbH, Isprung, Germany) under the following conditions: 60 °C for 10 h. The dimensions of the three different types of specimens obtained were according to the ISO 527-2, 178 and 179-1 standards, respectively, to guarantee suitable characterization of the materials in terms of tensile, flexural, and impact strength properties [21,22,23].

#### 2.3.2. Mechanical Properties

The biocomposites were mechanically characterized in terms of their tensile, flexural, and Charpy impact strength properties. Tensile and flexural properties were determined according to ISO 527-2 and 178 standards, respectively, using a 10 KS universal testing machine (Tinius Olsen, Redhill, UK). A Charpy Impact Meter Izod IMPats 2281 (Metrotec, Lezo, Spain) was used to determine the Charpy impact strengths of the different materials according to the ISO 179-1 standard.

#### 2.3.3. Structural and Morphological Analysis

FT-IR and XRD analyses were performed for the structural characterization of the manufactured biocomposites. A Tensor 27 FT-IR spectrometer (Bruker, MA, USA) with a resolution of 4 cm^−1^ was used in the spectral range of 400–4000 cm^−1^, again applying ATR methodology to obtain the FT-IR spectra of the different samples. A Bruker Platinum diamond glass ATR accessory with stainless steel glass holder and pressure control was used. FT-IR spectra were thoroughly analyzed to determine the main chemical bonds present in the biocomposites as well as the changes, in terms of chemical bonds, of the material as a consequence of the degradation process. The crystalline structure of the biocomposites was determined by XRD using the same equipment and methodology described in Section 2.1. In addition, the morphology of the fracture surface of the specimens previously subjected to Charpy trials was analyzed using SEM coupled with Energy Dispersive X-ray Spectroscopy (EDS) (JSM 840, JEOL, Tokyo, Japan).

The degradation of the material was quantified by means of the comparative analysis of their Carbonyl Indices (*CI*) calculated using Equation (1).

(1) $$CI=\frac{A_{1715}}{A_{974}}$$

where *A*_1715_ and *A*_974_ are, respectively, the absorbances at 1715 and 974 cm^−1^ of the samples analyzed, and which are due to the carbonyl groups present [24].

#### 2.3.4. Water Absorption

The water absorption capacity (*c*) was determined by immersion. Specimens with dimensions 80 mm × 10 mm × 4 mm were immersed in distilled water at 23 °C, as described in the ISO 69 standard [25]. The results obtained were expressed as the average (five replicates) percentage of the mass of water absorbed by the sample using Equation (2):

(2) $$c\left(\%\right)=\frac{m_{2}-m_{1}}{m_{1}}\times 100$$

where *m*_1_ is the initial mass of the specimen before immersion (mg), and *m*_2_ is the mass of the specimen after being subjected to the immersion process defined in the ISO 69 standard.

#### 2.3.5. Differential Scanning Calorimetry (DSC)

The thermal stability of the manufactured materials was determined by DSC using a Mettler Toledo 1 Stare System (Mettler Toledo, Barcelona, Spain). The crystallinity and thermal transitions were analyzed in the temperature range of −30 °C to 350 °C, with a heating rate of 5 °C/min and an ultra-high purity N_2_ flow rate of 50 mL/min. Aluminium crucibles with 40 µL capacity and a lid were used. The percentage of crystallinity (*W_c_*) was calculated using Equation (3):

(3) $$W_{c}\left(\%\right)=\frac{\Delta H_{m}}{\Delta H_{mc}}\times \frac{1}{f}\times 100$$

where Δ*H_m_* (J/g) is the enthalpy of fusion of the analysed material, calculated as the area contained in the section of the curve corresponding to the peak of the endothermic melting process; Δ*H_mc_* (J/g) corresponds to the enthalpy of fusion of 100% crystalline PLA (93.7 J/g) [26]; and *f* is the weight fraction of the polymer matrix (PLA or PLAD) in the biocomposite.

## 3. Results

### 3.1. OP Characterization

**Figure 1:**
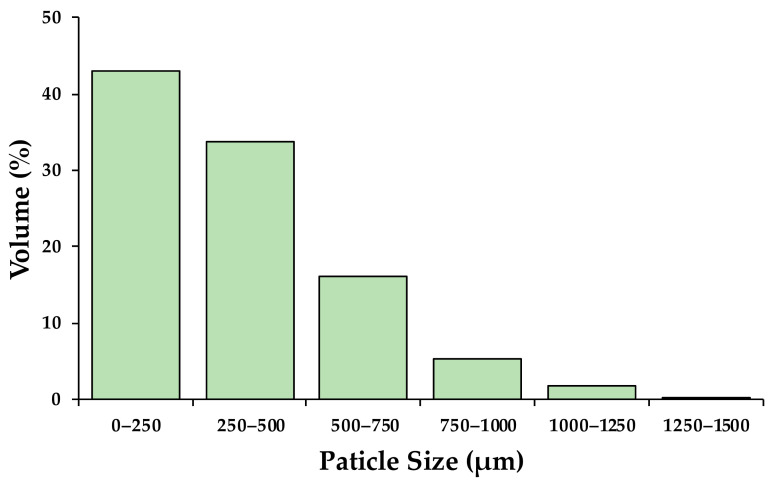
OP particle size distribution.

The particle size distribution of the conditioned OP samples used as reinforcements in PLA matrix is shown in Figure 1. The results showed that 43% of the granules obtained were less than 250 µm in size, maintaining 93.7% with a size less than 750 µm.

**Figure 2:**
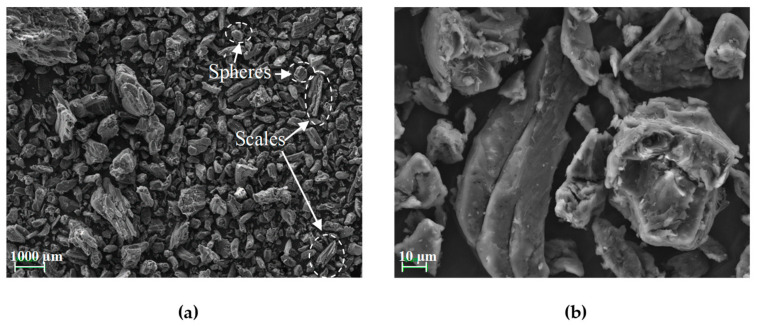
SEM images obtained from the OP particles after being subjected to the shredding process (**a**) 250 magnification; (**b**) 2000 magnification.

Images obtained from the morphological analysis of the OP particle surfaces at different magnifications are presented in Figure 2. These particles exhibit an irregular morphology typical of powders derived from food pits [6], with structures in the form of spheres and scales [20].

The moisture, ash, cellulose, hemicellulose, and lignin contents of the OP samples are listed in Table 4. These results show that the composition of OP was similar to that previously reported by other authors [6,27]. Valvez et al. (2021) found that OP is composed of 31% wt. cellulose, 22% wt. hemicellulose and 27% wt. lignin [28]. The remaining 15.72 wt.% until completing the total composition of the sample is formed by different chemical compounds in the extractive fraction, such as fats, proteins, free sugars, esters and others, as indicated previously [28,29].

**Figure 3:**
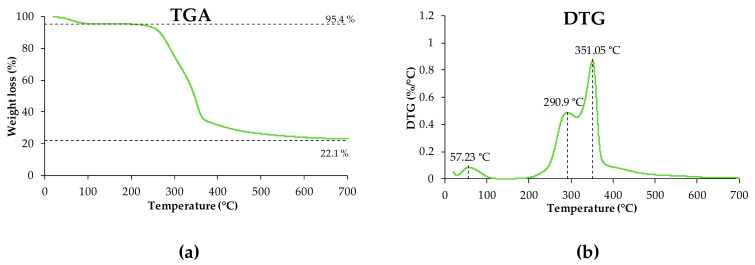
TGA (**a**) and DTG (**b**) curves resulting from the thermal analysis of the OP samples.

The results corresponding to the thermal properties of the OP obtained from the TGA curves are shown in Figure 3. A loss of 4.5% wt. was observed in the temperature range below 100 °C due to moisture and volatiles loss. As the temperature rises, the OP mass remains stable below 200 °C, indicating that this reinforcement can be processed in this temperature range without risk of degradation [7]. At higher temperatures, the degradation phase of the OP takes place, which is divided into two well-differentiated stages: (a) the first stage occurs until reaching 315 °C with peak at 291 °C that is mainly due to the degradation of hemicellulose and partially to lignin decomposition; and (b) the second stage, which takes place up to approximately 400 °C (peak at 351 °C) that mainly corresponds to the degradation of the cellulose fraction, but also partially to lignin. From 400 °C onwards, lignin continues to decompose until leaving a residue of approximately 22.1% wt. at 700 °C.

According to the study by Yang et al. (2007), the thermal decomposition of hemicellulose mainly occurs in the range 220–315 °C, whereas cellulose degrades between 315 °C and 400 °C. However, the thermal degradation of lignin occurs in a wider temperature range (160–900 °C) since it exhibits slow degradation kinetics [30]. These findings perfectly match with the degradation pattern of olive pits observed in Figure 3. It is shown in this figure that most weight loss (~73% wt.) occurred between approximately 160 °C and 400 °C, thus reflecting the lignocellulosic nature of OP.

### 3.2. Mechanical Properties of Biocomposites

Table 5 lists the results obtained for the different parameters analyzed in the tensile tests: maximum tensile stress (σ_m_), tensile stress at break (σ_b_), maximum tensile strain (ε_m_), yield strain at break (ε_b_) and Young’s modulus (E_t_). In general, the introduction of OP into the PLA matrix had a very significant influence on the mechanical properties of the manufactured biocomposites. σ_m_ decreases when incorporating OP, this effect being greater as the percentage by weight of OP in the material increases, going from 53.16 MPa for PLA to 11.92 MPa for PLA-40OP, that is, a decrease of 77.6%. The behavior of σ_b_ is very similar due to the lack of compatibility between the OP and the polymer matrix, which gives rise to the agglomeration phenomena of small-sized OP particles that become stress concentrators [7,31]. A decrease in the values of ε_m_ was also observed, going from 6.45% (PLA) to 1.66% (PLA-40OP), which indicates that the incorporation of OP gives rise to increased brittleness. Nevertheless, certain percentages of OP in the material generated an increase in E_t_, which was almost 30% higher than that of pure PLA when 15% OP was incorporated into the PLA matrix.

After the degradation process, a slight decrease in practically all the tensile properties of the PLA was observed. Karamanlioglu and Alkan (2019) [32] also reported a decrease in all the tensile properties after exposure of PLA at room conditions (20 °C and 40% relative humidity), and high temperature and low humidity. This decrease, of up to 67.9%, 91.8% and 24.4% for σ_m_, ε_m_ and E_t_, respectively, was assumed to be due to hydrolysis of ester bonds, especially in the amorphous regions of PLA, which governs the degradation mechanism of PLA by environmental exposure [32]. In the biocomposites manufactured in this study, the decrease in E_t_ as a consequence of the degradation process also occurred. Nevertheless, there was an increase in σ_m_ in PLAD-based biocomposites compared to PLA-based biocomposites, which was due to the higher crystallinity of the biocomposites after the degradation process [33].

Table 6 shows the values of the parameters obtained from the flexural tests performed: maximum flexural strength (σ_fm_), flexural strength at the breaking point (σ_fb_), deformation at the point of maximum flexural strength (ε_fm_), flexural strain at the breaking point (ε_fb_), and flexural modulus (E_f_). The incorporation of OP particles had an effect on the flexural behavior of the materials, similar to the effect on the tensile behavior; that is, it produced a decrease in the values of flexural strength and deformation, this effect being greater as the OP percentage increases. The values of σ_fm_ and ε_fm_ decreased by up to 56.4% and 64%, respectively, compared to those of pure PLA with 40% wt. of OP. This behavior is attributed to poor interfacial adhesion between the hydrophilic reinforcement and the hydrophobic polymer matrix [7]. The increase in OP content implies that there was more OP surface that needed to be in contact with the polymeric matrix to generate a good interaction between the phases, leading to an insufficiently efficient stress transfer, which means that poor dispersion and the formation of agglomerates cause a decrease in properties [34]. Furthermore, since pure PLA is a brittle material, the addition of OP particles that behave as fillers causes an increase in its brittleness [28]. Nevertheless, there was also an increase in the value of the flexural modulus, which increased as the percentage of OP increased, reaching an increase of up to 25.5% for PLA-40OP compared to pure PLA. This is mainly due to the fact that the incorporation of OP increases the rigidity of the final biocomposite [35]. Finally, the degradation of PLA did not imply a significant variation in the flexural properties of the biocomposites, with pure PLAD exhibiting higher flexural strength and lower flexural modulus as compared with PLAD/OP composites.

**Figure 4:**
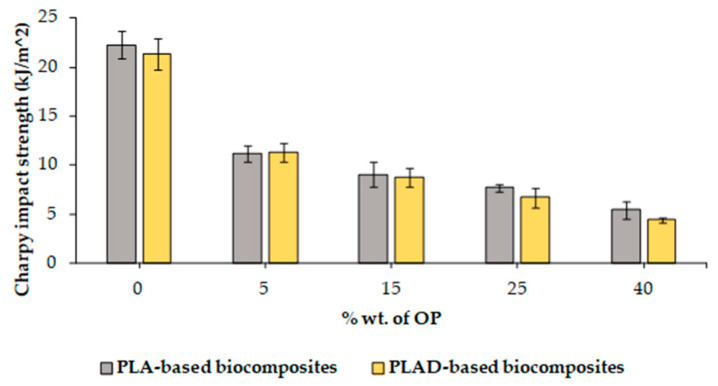
Charpy impact strength properties of PLA-based and PLAD-based biocomposites.

Figure 4 shows a comparison of the values obtained from the Charpy impact strength tests carried out on the different materials. The results indicate that the incorporation of OP particles decreased the impact resistance of the manufactured biocomposites, reaching up to 49.8% when 5% wt. of OP is introduced. This effect has been previously reported by other authors, and mainly occurs due to a lack of adherence between phases [28]. The degradation of the PLA resulted in a slight decrease in the worst case, both in the pure polymer matrix and biocomposites. This is in line with the decrease in tensile and flexural strengths.

The decrease in the mechanical properties of OP composites with respect to pure PLA has been observed when other thermoplastic matrices such as polystyrene [8], recycled post-consumer plastic [9], polypropylene [10,11] or poly(ε-caprolactone) [12], were used. The possible causes of this decrease may be: (i) the hydrophilic nature of the OP that absorb more moisture [8], (ii) poor adhesion with the non-polar matrix [9,12], (iii) agglomeration of OP particles [10,11], or (iv) limited mobility of the polymer chains [12]. In the case of PP, the use of compatibilizers, such as MAPP, reduces the decrease in properties by improving the cohesion between the phases [10,11].

### 3.3. Fourier Transform Infrared Spectroscopy (FT-IR)

**Figure 5:**
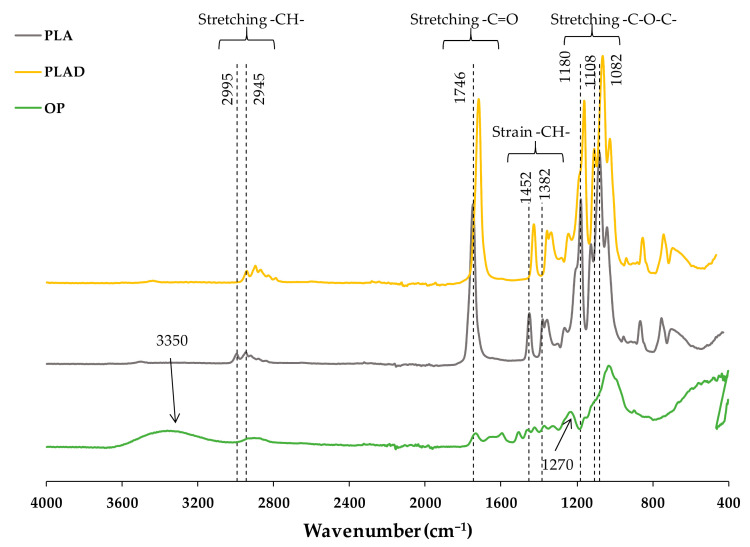
FT-IR spectra of OP, PLA, and PLAD.

Figure 5 shows the FT-IR spectra obtained for the OP, PLA-based biocomposite and PLAD-based biocomposite samples. In the OP FT-IR spectrum, the peaks corresponding to the functional groups typical of lignocellulosic materials stand out. A first band was identified around 3350 cm^−1^, corresponding to –OH groups. A second, of medium intensity, is located around 1270 cm^−1^, which is associated with the stretching of the C-H bonds. Both bands identified the functional groups present in cellulose [36]. The strong band at 1089 cm^−1^ was attributed to the vibration of the glycosidic bond (C-O-C) superimposed with the stretching vibrations of the C-OH bond present in cellulose, hemicelluloses, and lignin [37].

The PLA spectrum identified bands associated with the most representative functional groups of the polymer matrix: (a) 2995 and 2945 cm^−1^, stretching of the CH group; (b) 1746 cm^−1^, stretching of the C=O group [38]; (c) 1452 and 1382 cm^−1^, deformation of the CH bond; and (d) 1452 cm^−1^, a peak generally associated with the presence of lactic acid [32]. The peaks located at 1180, 1108 and 1082 cm^−1^ were attributed to the stretching of –C-O-C. The absence of bands in the region attributed to –OH bonds (around 3350 cm^−1^) was also observed, indicating that the number of –OH groups present in the material was very poor [38]. The peaks located at 875 and 756 cm^−1^ were attributed to the amorphous and crystalline regions of the polymer, respectively [32].

The FT-IR spectrum of PLAD showed a significant increase in the intensity of the peaks located at (a) 1180 and 1082 cm^−1^, due to the stretching of the ester bond, and (b) at 1750 cm^−1^, attributed to the stretching of the carbonyl group [37]. The peak located at 875 cm^−1^ decreased in amplitude because degradation began in the amorphous region, increasing the crystallinity of the final material. Although the PLA samples were under environmental conditions, the FT-IR of the PLAD indicated signs of hydrolysis of the ester bonds, which is in agreement with what was reported by other authors who indicated that, in the degradation process of semicrystalline PLA, the ester bonds in amorphous regions were initially affected [32].

**Figure 6:**
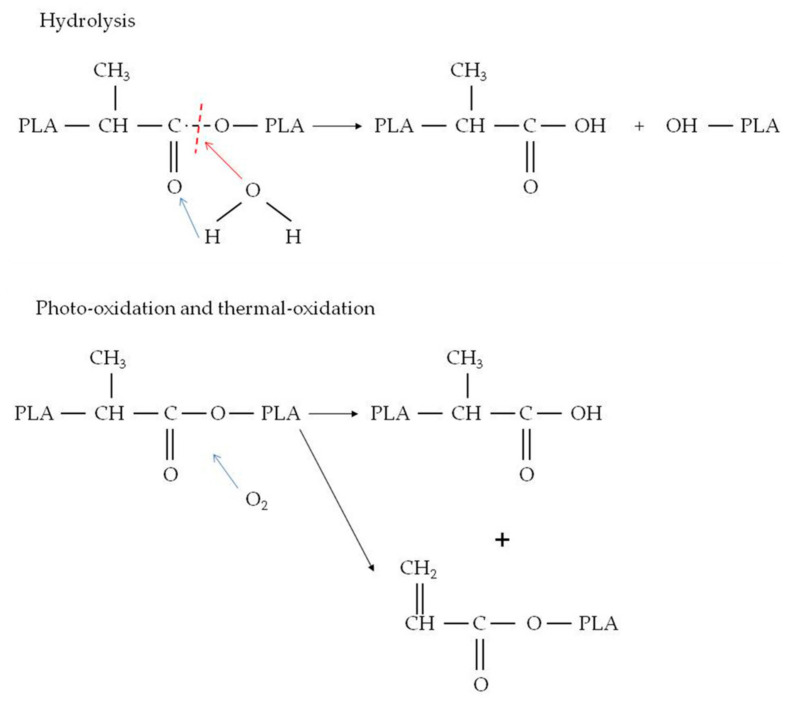
PLA degradation mechanisms.

The PLA degradation mechanisms are shown in Figure 6, which depicts the breakdown of PLA macromolecules through ester bond breaking or production of smaller molecules [39]. Before the degradation of the material, the lignocellulosic fillers guaranteed entry for moisture penetration by capillary action, owing to the hydrophilic tendency [2].

**Figure 7:**
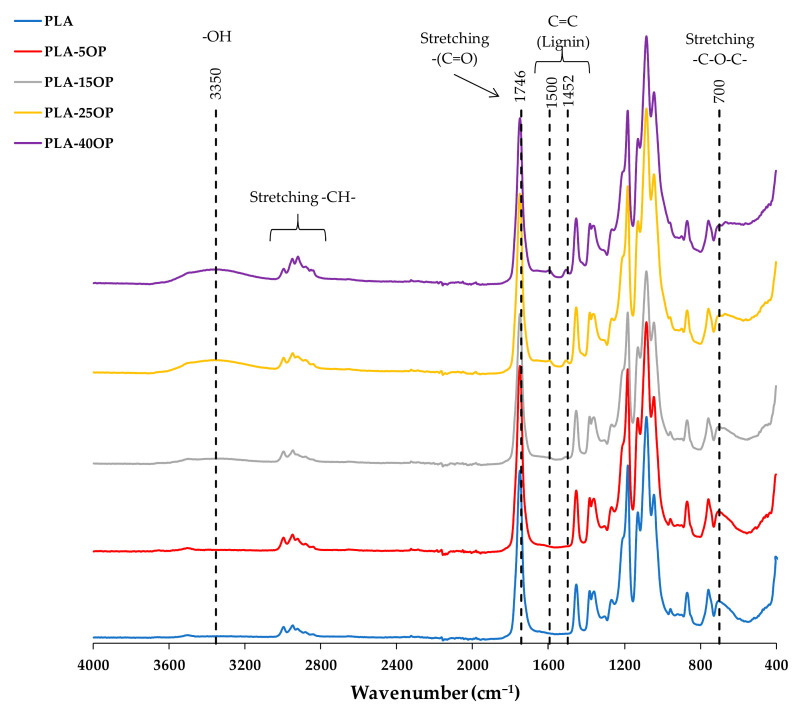
PLA-based biocomposite FT-IR spectra.

**Figure 8:**
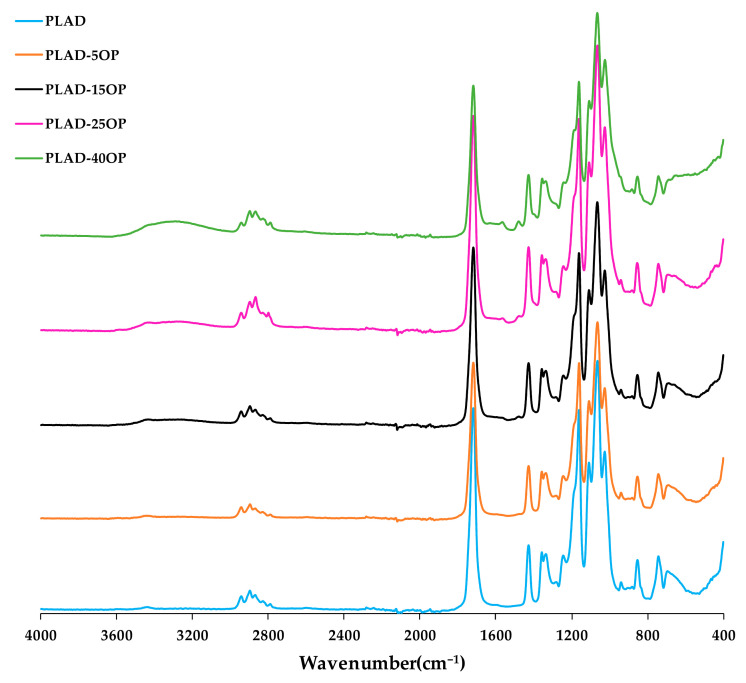
PLAD-based biocomposite FT-IR spectra.

Figure 7 and Figure 8 show the spectra of the PLA-based and PLAD-based biocomposites, respectively, including those of the pure matrices (PLA and PLAD). In the materials containing OP, it was clearly observed that there was a superposition of the bands present in the most representative regions of PLA and the lignocellulosic component and that the molecular structure of PLA was maintained, indicating that the mixture of materials was a physical binding process [31]. The appearance of the band corresponding to the –OH groups indicates a greater presence of free –OH groups; this band is stronger when the OP content increases [6,38]. This band was not observed for the spectrum obtained for pure PLA (Figure 5). The bands present in the region located between 3000 and 2800 cm^−1^ are representative of the -CH group present in cellulose, hemicellulose, and lignin. The band around 700 cm^−1^ is associated with the stretching vibrations of the C-O group present in lignin [6]. In addition, peaks near 1452 and 1500 cm^−1^ were observed due to the vibration of the carbon skeleton (C=C) of the benzene ring on the molecular structure of the lignin present in the OP [31].

Both the PLA-based and PLAD-based biocomposite spectra present broad bands located between 3100 and 3600 cm^−1^, which are associated with the -OH stretching vibrations of the OP reinforcement used [6]. The increased presence of hydroxyl groups in biocomposites, owing to the presence of OP, can promote hydrophilicity and water absorption, accelerating the degradation of the materials [31,36]. Moreover, the appearance of these peaks corresponding to OP may be due to PLA not completely encapsulating the OP particles [31].

**Figure 9:**
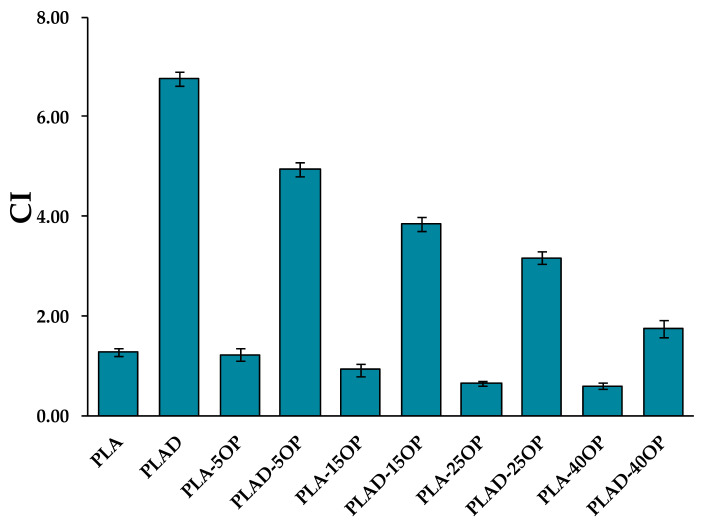
CI of PLA-based and PLAD-based biocomposites.

The CI was calculated as a measure of the degree of degradation of the PLA and biocomposites. Figure 9 shows the CI obtained for all the samples before and after the degradation. Non-degraded samples did not show CI of 0 because the PLA structure contains C=O bonds even when the material is not degraded. The CI values of the undegraded samples were found to be much lower than those of the degraded samples. As the percentage by weight of OP in the biocomposites increased, the CI value decreased, this decrease being much more notable in the case of materials subjected to degradation. Generally, the PLA decomposition mechanism produces a greater number of decomposition products (intermediate and/or final) with C=O bonds in their chemical structures [17]. On the contrary, the data obtained indicate that the presence of OP produced fewer C=O groups. This result was probably due to the presence of OP preventing the passage of O_2_ and therefore the formation of new C=O groups [40].

### 3.4. X-ray Diffraction (XRD)

**Figure 10:**
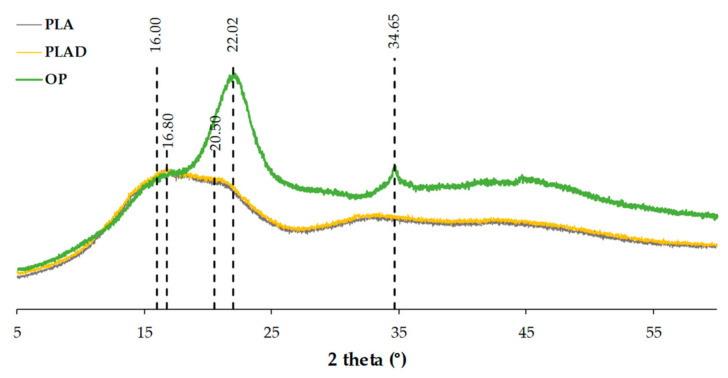
Diffraction patterns obtained for OP, PLA and PLAD.

Figure 10 shows the diffraction patterns of the OP, PLA and PLAD samples. The diffraction pattern of OP shows three main peaks at 16°, 22°, and 34.6° corresponding to the (110), (200) and (004) crystallographic planes, respectively, which are typical of lignocellulosic materials due to the presence of cellulose type I crystals [37,38,41]. The pure PLA presents a wide region, centered at 20.5° and with a slight intensity peak at 16.8°, corresponding to the α’ [42] crystallization phase, indicating that amorphous PLA was used [34,38].

**Figure 11:**
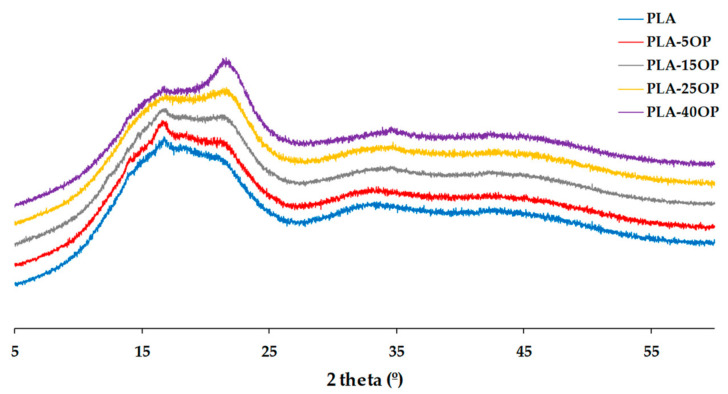
Diffraction patterns obtained for PLA-based biocomposites.

**Figure 12:**
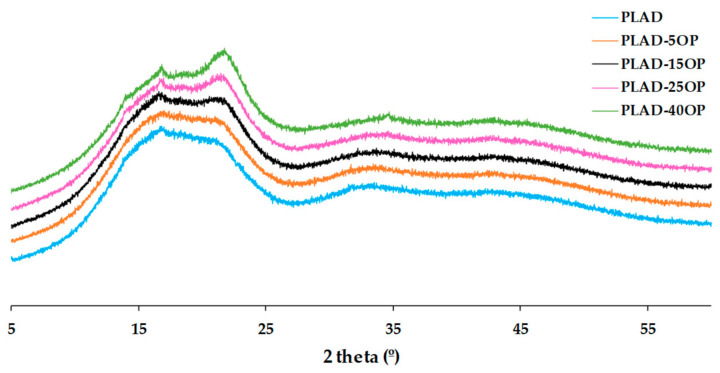
Diffraction patterns obtained for PLAD-based biocomposites.

Figure 11 and Figure 12 show the diffraction patterns obtained from the PLA-based and PLAD-based biocomposites, respectively, including the pure polymer matrices. The introduction of OP increased the intensity of the diffraction peak associated with the (200) plane, corresponding to cellulose, accentuating this effect when increasing the percentage of OP [34]. The peaks of the PLAD-based biocomposites are slightly more pronounced because the degradation process removes part of the polymeric matrix, leaving the OP particles more accessible. However, the peaks corresponding to OP were similar in both types of biocomposites because of the change in crystallinity after the degradation process [42]. There is also a broadening effect at 2θ = 16.5° in the patterns of the PLAD-based biocomposites, indicating that OP favors the absorption of water and therefore PLA degradation [43].

### 3.5. Scanning Electron Microscopy (SEM)

**Figure 13:**
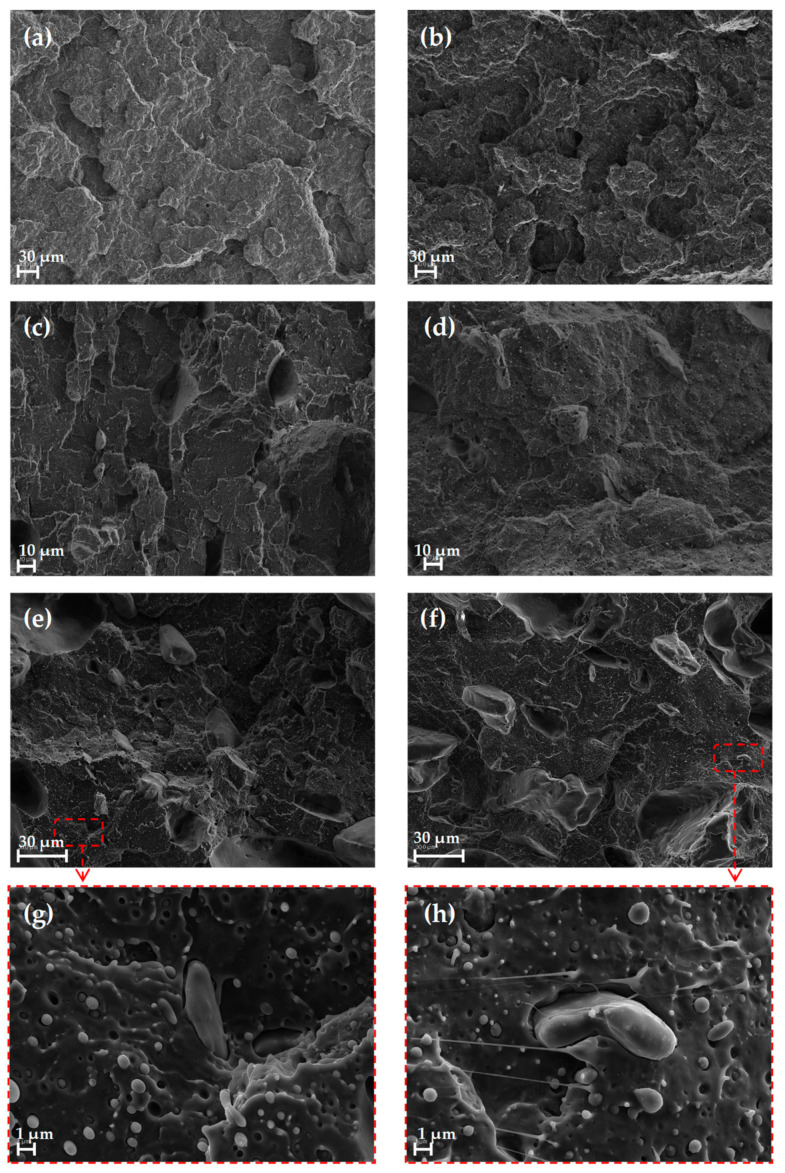
SEM images of the fracture surface of the different types of tensile samples tested: (**a**) PLA; (**b**) PLAD; (**c**) PLA-5OP; (**d**) PLAD-5OP; (**e**) PLA-25OP; (**f**) PLAD-25OP; (**g**) PLA-5OP; (**h**) PLAD-5OP.

Figure 13 shows the fracture surfaces of the specimens tested in terms of Charpy impact strength. In Figure 13a, the fracture surface corresponds to a pure PLA specimen, which can identify a type of brittle fracture [31]. In the case of the PLAD samples, a greater presence of a layered texture was observed (Figure 13b), possibly due to the influence of degradation.

In the case of the materials that incorporated OP, the presence of particles mixed with the polymer matrix was observed (Figure 13c,e), as well as numerous traces corresponding to its extraction after the impact test [31,37]. In general, all the materials analyzed presented practically the same topology on the fracture surface, although tensile stress due to OP was detected in the biocomposites [2]. These cracks, together with the capillarity of the material components themselves, favor water molecules attacking the matrix-filler interface, resulting in a more pronounced separation between the polymer matrix and the OP particles [2]. OP particles are presented in the form of granules, which often have drawbacks related to the phase boundary and increasing cavity spaces [6]. In Figure 13g,h, a lack of interfacial adherence between the OP particles and PLA matrix can be observed, giving rise to a low stress transfer and corroborating the loss of mechanical properties [7,37].

### 3.6. Water Absorption

**Figure 14:**
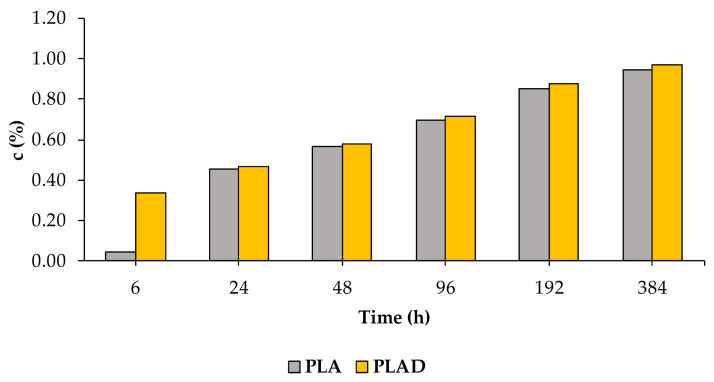
Values of *c* for PLA and PLAD.

Figure 14 shows the variation in the percentage of the mass of water absorbed by the PLA and PLAD test specimens. From the curves, two stages can be clearly distinguished in the water absorption process: (a) the first 24 h of immersion and (b) the period that elapses between 24 h and 384 h of immersion. PLAD absorbed much more water than PLA during the first stage, while the trend evened in the second stage, with c values for PLAD being slightly higher than those for PLA [4]. A similar behavior was reported by Chen et al. [4] for PLA/jute biocomposites.

**Figure 15:**
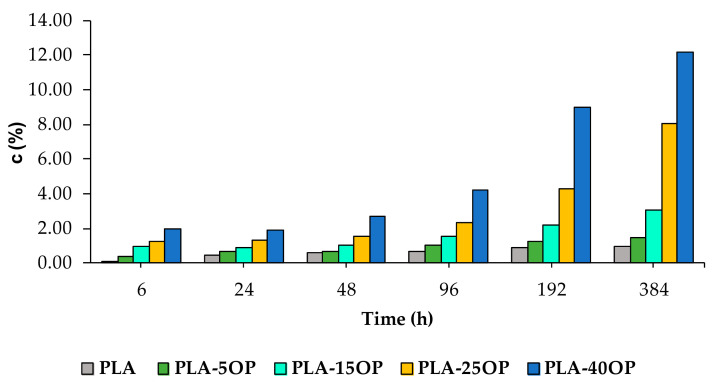
Values of *c* for the PLA-based biocomposites.

**Figure 16:**
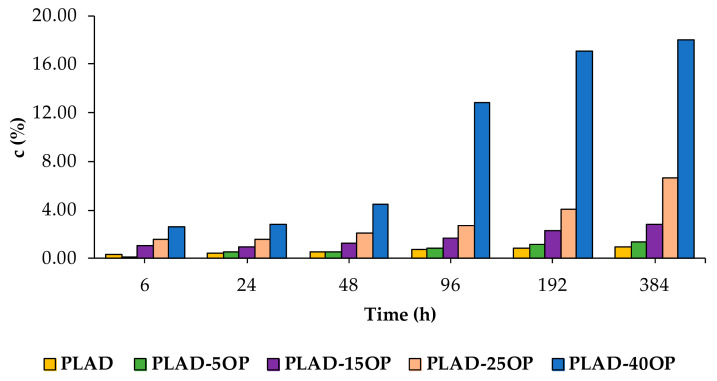
Values of *c* for the PLAD-based biocomposites.

Figure 15 and Figure 16 show the water absorption of PLA-based and PLAD-based biocomposites, respectively, including PLA and PLAD. The water absorption of the PLA-based biocomposites was greater than that of pure PLA, indicating that the presence of OP increased the adhesion of water molecules, thereby increasing the hydrophilic nature of the material [4,36]. Pure PLA showed a mass variation of 0.95% after 384 h of immersion, compared to the 12.14% variation presented by PLA-40OP. This mass variation was greater as the OP percentage increased and as the immersion time increased owing to the presence of holes in the interface, corroborating the SEM images obtained from the fracture surface (Figure 13). The same was previously reported by other authors who incorporated jute fibers into a PLA matrix [4].

PLAD-based biocomposites reported higher water absorption values of up to 18% for the PLAD-40OP sample compared with 12.14% for the PLA-40OP sample.

In composites of OP with other polymer matrices such as PVC [10], the same behavior is observed. This is a consequence of the hydrophilic nature of the filler associated with the existence of voids, pores and cracks in the filler/matrix interface that favor the diffusion of water by capillary effect.

### 3.7. Differential Scanning Calorimetry (DSC)

**Figure 17:**
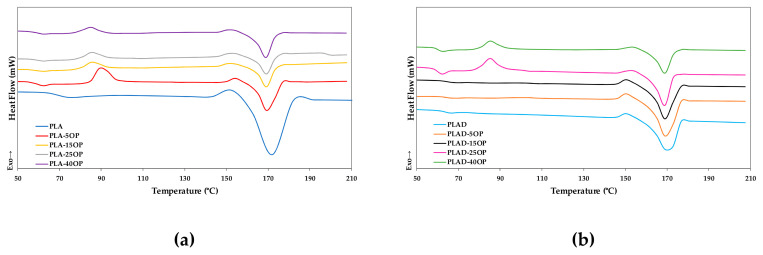
DSC curves of (**a**) PLA-based biocomposites and (**b**) PLAD-based biocomposites.

Figure 17 shows the DSC curves of the PLA-based and PLAD-based biocomposites, including PLA and PLAD. The DSC curves of the PLA base materials presented two endothermic signals corresponding to the glass transition (65 °C) and melting of the material (171.4 °C) [44]. The introduction of OP in high percentages favored the appearance of an endothermic crystallization peak, influencing the reorganization process of the amorphous chains in both PLA-based and PLAD-based materials [45].

Table 7 shows the glass transition temperature (*T_g_*), crystallization temperature (*T_c_*), melting temperature (*T_m_*), Δ*H_m_*, and *W_c_* of all the materials studied. When OP particles were introduced, there was a decrease in the *W_c_* values of the PLA-based biocomposites with respect to pure PLA. This was because the OP hindered the mobility of the PLA chains, preventing the formation of crystals [7,44] from a *W_c_* value of 47% for pure PLA to 30.42% for PLA-15OP. A similar effect was observed for PLAD-based materials. The hydrolytic process, initiated when the diffusion of water to the amorphous zones occurs, also contributed significantly to the increase in *W_c_* of PLAD with respect to PLA [46]. Regarding biocomposites, PLAD-based biocomposites also presented higher *W_c_* values than PLA-based biocomposites, which is not only due to hydrolytic degradation but also to its synergistic effect with the OP particles that allows the alignment of the PLA chains and their rearrangement into a more stable crystalline structure [47]. PLAD-based materials showed a slight decrease in *T_g_* and *T_m_* compared to PLAD-based materials. This decrease was due to the loss of molecular weight after degradation [31]. This effect was not significant, but it has been previously reported by other authors who studied the effect on the thermal properties of PLA after its immersion in water [48]. Finally, *T_c_* shifted towards lower temperatures due to chain scission [47].

## 4. Conclusions

The introduction of OP into the PLA matrix affected the mechanical properties of the manufactured biocomposites, similarly to other biocomposites reinforced with OP using a different polymer matrix. The tensile strength and elongation decreases when OP was incorporated; nevertheless, an increase in *E_t_* of almost 30% was observed. The degradation led to significant decreases in the tensile properties of the biocomposites; nevertheless, an increase in *σ_m_* occurred because of the increase in the crystallinity of the biocomposites. The trend in the behavior of the materials in terms of flexural properties turned out to be similar to their tensile behavior. As for the impact resistance, there was an even more noticeable decrease due to the lack of adhesion between the phases and degradation.

The FT-IR spectra and diffraction patterns confirmed the degradation and changes in the crystalline structure of the materials originating as a consequence of the storage conditions. The analysis of the CI values indicated that the degree of degradation of the biocomposites was much greater than that of virgin PLA; however, the degradation of the biocomposites reached a lower degree as the percentage of OP increased, probably because the OP prevented the flow of O_2_ and therefore the formation of new C=O groups.

SEM images of the fracture surface of the impact-tested samples confirmed the increased presence of a layered texture in the degraded materials and the lack of interfacial bonding, resulting in low stress transfer and corroborating the loss of mechanical properties.

PLAD presented a significantly higher *c* than PLA in the first 24 h, equilibrating from that moment on in the following hours of immersion. In turn, virgin materials showed less c than biocomposites, with c being higher as the percentage by weight of OP increased, which confirmed that OP favors water absorption. PLAD-based biocomposites reported higher *c* values than PLA-based biocomposites, with the influence of degradation being important, although less significant than OP weight percentage.

The OP particles caused a decrease in *W_c_* of the PLA-based biocomposites with respect to pure PLA, mainly due to the greater difficulty of movement of the PLA chains. A similar effect was observed for PLAD-based materials. The hydrolytic process also contributed significantly to the increase in *W_c_* of PLAD with respect to PLA. PLAD-based biocomposites also presented higher *W_c_* values than PLA-based biocomposites, which was not only due to hydrolytic degradation but also to its synergistic effect with OP particles, allowing the alignment of the PLA chains and their rearrangement into a more stable crystalline structure. PLAD-based materials showed a slight decrease in *T_g_* and *T_m_* compared to PLAD-based materials due to molecular weight loss after degradation. *T_c_* was shifted towards lower temperatures due to chain scission.

## Figures and Tables

**Table 1:** Composition of manufactured PLA-based biocomposites.

Reference	PLA (% wt.)	OP (% wt.)	BYK (% wt.)	PO1020 (% wt.)
PLA	94.5	-	1.5	4
PLA-5OP	89.5	5	1.5	4
PLA-15OP	79.5	15	1.5	4
PLA-25OP	69.5	25	1.5	4
PLA-40OP	54.5	40	1.5	4

**Table 2:** Denomination and composition of PLAD-based biocomposites.

Reference	PLA (% wt.)	OP (% wt.)	BYK (% wt.)	PO1020 (% wt.)
PLAD	94.5	-	1.5	4
PLAD-5OP	89.5	5	1.5	4
PLAD-15OP	79.5	15	1.5	4
PLAD-25OP	69.5	25	1.5	4
PLAD-40OP	54.5	40	1.5	4

**Table 3:** Compounding process parameters.

Temperature (°C)							Screw Rotation Speed (rpm)
Zone I	Zone II	Zone III	Zone IV	Zone V	Zone VI	Nozzle	
155	160	170	170	170	165	165	175

**Table 4:** Composition of OP samples.

Moisture (% wt.)	Ash (% wt.)	Cellulose (% wt.)	Hemicellulose (% wt.)	Lignin (%wt.)
5.11	0.00	21.79	23.60	33.78

**Table 5:** Tensile properties of PLA-based biocomposites and PLAD-based biocomposites.

Reference	σ_m_ (MPa)	σ_b_ (MPa)	ε_m_ (%)	ε_b_ (%)	E_t_ (MPa)
PLA	53.16 ± 0.67	37.47 ± 2.08	6.45 ± 0.22	13.12 ± 0.64	3523.96 ± 166.61
PLA-5OP	29.45 ± 0.94	23.88 ± 0.79	3.37 ± 0.21	6.56 ± 0.34	3525.85 ± 231.96
PLA-15OP	27.83 ± 0.77	24.90 ± 0.72	2.53 ± 0.14	3.91 ± 0.20	4578.21 ± 160.75
PLA-25OP	20.89 ± 0.42	19.22 ± 0.50	2.59 ± 0.14	2.59 ± 0.14	3491.67 ± 293.07
PLA-40OP	11.92 ± 0.06	10.65 ± 0.42	1.66 ± 0.12	1.66 ± 0.12	2244.43 ± 73.63
PLAD	51.59 ± 0.71	43.92 ± 2.08	5.96 ± 0.21	7.53 ± 0.82	3464.00 ± 315.02
PLAD-5OP	44.30 ± 0.36	41.37 ± 1.05	4.41 ± 0.21	5.39 ± 0.52	2995.36 ± 298.66
PLAD-15OP	32.06 ± 1.52	32.08 ± 1.30	3.08 ± 0.18	3.20 ± 0.19	3197.40 ± 156.35
PLAD-25OP	22.80 ± 2.32	22.99 ± 2.34	2.29 ± 0.48	2.39 ± 0.49	2807.83 ± 134.40
PLAD-40OP	13.30 ± 0.43	13.27 ± 0.40	1.09 ± 0.19	1.19 ± 0.20	2754.06 ± 111.40

**Table 6:** Flexural properties of PLA-based biocomposites and PLAD-based biocomposites.

Reference	σ_fm_ (MPa)	σ_fb_ (MPa)	ε_fm_ (%)	ε_fb_ (%)	E_f_ (MPa)
PLA	70.36 ± 0.56	68.82 ± 0.92	3.72 ± 0.19	5.00 ± 0.00	2667.99 ± 8.37
PLA-5OP	56.34 ± 0.82	45.10 ± 0.67	2.24 ± 0.09	3.02 ± 0.50	2901.59 ± 74.94
PLA-15OP	52.23 ± 1.27	42.75 ± 1.68	1.90 ± 0.12	2.16 ± 0.30	3324.44 ± 114.80
PLA-25OP	43.69 ± 2.78	35.49 ± 2.41	1.80 ± 0.29	2.16 ± 0.50	3348.53 ± 111.36
PLA-40OP	30.66 ± 0.46	24.65 ± 0.19	1.34 ± 0.05	1.47 ± 0.10	3349.44 ± 68.92
PLAD	71.24 ± 0.71	57.05 ± 0.75	3.70 ± 0.16	4.47 ± 0.13	2855.94 ± 20.74
PLAD-5OP	62.80 ± 0.58	52.10 ± 4.25	3.69 ± 0.29	4.85 ± 0.17	2913.71 ± 46.15
PLAD-15OP	49.47 ± 1.13	39.74 ± 1.02	2.34 ± 0.11	2.67 ± 0.13	3131.80 ± 39.62
PLAD-25OP	36.61 ± 0.92	29.54 ± 0.79	1.70 ± 0.07	1.95 ± 0.13	3205.85 ± 35.07
PLAD-40OP	25.76 ± 0.22	13.52 ± 3.11	1.10 ± 0.00	1.55 ± 0.22	3477.69 ± 51.67

**Table 7:** Thermal properties of PLA-based biocomposites and PLAD-based biocomposites.

Reference	*T_g_* (°C)	*T_c_* (°C)	*T_m_* (°C)	Δ*H_m_* (J/g)	*W_c_* (%)
PLA	65.07	-	171.42	41.62	47.00
PLA-5OP	61.05	90.47	170.69	31.10	37.09
PLA-15OP	61.42	86.13	170.56	22.66	30.42
PLA-25OP	61.40	85.85	170.54	25.23	38.74
PLA-40OP	62.09	84.53	170.16	26.23	51.37
PLAD	61.47	-	171.02	47.43	53.56
PLAD-5OP	60.23	-	170.56	41.02	48.91
PLAD-15OP	62.33	-	170.41	39.16	52.57
PLAD-25OP	58.39	85.04	169.98	33.44	51.34
PLAD-40OP	60.38	85.54	170.33	24.62	48.21

## Data Availability

The data presented in this study are available on request from the corresponding author.
